# Evolutionary Patterns Under Climatic Influences on the Distribution of the *Lycoris aurea* Complex in East Asia: Historical Dynamics and Future Projections

**DOI:** 10.3390/plants15020272

**Published:** 2026-01-16

**Authors:** Weiqi Meng, Xingshuo Zhang, Haonan Zhang, Guoshuai Hou, Lianhao Sun, Xiangnan Han, Kun Liu

**Affiliations:** 1 Innovative Research Team for Forest Restoration Mechanisms, Chishui National Ecological Quality Comprehensive Monitoring Stations, Nanjing Institute of Environmental Sciences, Ministry of Ecology and Environment (MEE), Nanjing 210042, China; 2 College of Wildlife and Protected Area, Northeast Forestry University, Harbin 150040, China; 3 Co-Innovation Center for Sustainable Forestry in Southern China, Nanjing Forestry University, Nanjing 210037, China; 4 Anhui Provincial Key Laboratory of the Conservation and Exploitation of Biological Resources, College of Life Sciences, Anhui Normal University, Wuhu 241000, China

**Keywords:** *Lycoris aurea*, MaxEnt, distribution pattern, cytotype, divergence

## Abstract

Investigating plant responses to climate change is critical for understanding phylogeography and devising conservation strategies. This study focuses on the *Lycoris aurea* (L’Hér.) Herb. complex in East Asia, a system characterized by high cytotype diversity (2n = 12–16), to test whether ecological niche differentiation drives its spatio-temporal evolution. We integrated dynamic niche modeling to reconstruct distribution dynamics from the Last Interglacial (LIG) to the future (2100). Results indicate that mainland China populations have expanded northward since the LIG, establishing their current patterns, while island populations (Taiwan, Ryukyu) remained relatively stable due to geographic constraints. Under future warming scenarios, the complex is projected to further expand northward. We identified key migration corridors, with high inter-cytotype connectivity in the Sichuan-Hubei region and intra-cytotype migration in the Yunnan Plateau and Nanling region. Although the two dominant cytotypes currently exhibit niche equivalency, they show distinct climatic sensitivities—Cytotype II is driven by precipitation and Cytotype IV by temperature—and are projected to diverge spatially in the future. These findings elucidate the evolutionary history of *L. aurea* and provide a reference for the conservation and utilization of Lycoris germplasm.

## 1. Introduction

Climate change is a significant driver of biological evolution and distribution. Since the Quaternary glaciation, Earth’s climate has undergone drastic changes, particularly the cyclic advances and retreats of ice ages, leading to periodic sea level fluctuations and the recurrent formation and disappearance of land bridges between continents and islands [1]. In the past decades and even centuries, significant global climate change has been observed, with a warming trend. The Sixth IPCC Assessment Report predicts that the global surface temperature increase will exceed 1.5 °C between 2021 and 2040 [2]. Studies have shown that geological events such as continental drift, island formation, land bridge connections, and orogeny significantly impact species richness, distribution patterns, phenotypic morphology, and survival habits [3,4]. For most plants, global warming increases the environmental suitability of high latitudes and altitudes, leading species to expand or migrate to these more suitable areas. During glacial periods, global temperature declines cause species to contract towards lower altitudes and latitudes, seeking refugia [5,6,7,8].

Plant responses to climate change are a key issue in phylogeography and conservation biology [6]. Species distribution models (SDMs) are vital tools for studying species’ responses to climate change, predicting potential distribution areas, inferring migration routes and glacial refugia. Various models such as GARP, Bioclim, CLIMEX, and MaxEnt have been developed using different algorithms by scholars worldwide [9,10]. Among these, the MaxEnt model is widely used. It is based on known species distribution data and related environmental data, using specific algorithms to construct models and project results into different times and spaces to predict species’ distribution patterns. The model has been extensively applied in endangered species conservation, invasive species and pest control, and research on species origin and evolution [11,12,13].

To clarify the origin and evolutionary mechanisms of a species, a primary prerequisite is to identify its current distribution pattern and the driving factors behind its formation. However, species delimitation has always been a complex issue in plant taxonomy. Hong (2016) proposed the morphological-biological species concept, emphasizing consideration of multiple aspects such as morphological character variation, phylogenetic relationships, and reproductive isolation, and cited the case of the *Paeonia delavayi* complex [14]. A species complex is generally defined as a group of organisms that are morphologically cryptic but harbor significant biological differences, such as in their genetics, ecology, or cytotype. The formation of plant complexes is often the result of adaptive divergence, whereby populations adapt to heterogeneous environments, diverse climatic conditions, or different life strategies. This complex process involves a multifaceted interplay of ecological, physiological, and evolutionary factors. Tracing these interactions is therefore crucial for understanding the mechanisms of speciation, contemporary distribution patterns, and adaptive morphological evolution within these groups [15,16,17].

*Lycoris aurea* (L’Hér.) Herb. is a perennial herbaceous geophytic flowering plant belonging to the genus *Lycoris* in the Amaryllidaceae family, mainly distributed in East Asia, particularly in most regions south of the Yangtze River and Taiwan Province in China, the Ryukyu Islands, and Kyushu Island of Japan. It has high ornamental and medicinal value. Based on chromosome morphology and excluding hybrids, five karyotypes of *L. aurea* have been reported, contained 2n = 12 = 10m + 2T, 2n = 13 = 9m + 4T, 2n = 14 = 8m + 6T, 2n = 15 = 7m + 8T, and 2n = 16 = 6m + 10T [18]. Due to minimal morphological differences, it includes three infraspecific taxa: the typical variety *L. aurea* var. *aurea*, and the varieties *L. aurea* var. *surgens* and *L. aurea* var. *angustitepala*. *L. traubii* was published as a new species in 1957 [19]. It has three karyotypes (2n = 12, 13, 14) and is mainly distributed in Taiwan Province of China, the Ryukyu Islands, and islands such as Kyushu and Shikoku in Japan. This taxon emerges leaves in autumn without leaf base residues, and all other characteristics are similar to those of *L. aurea* distributed in mainland China [19,20,21,22,23]. Referring to Hong’s definition of the morphological-biological species concept, there is currently insufficient evidence to prove that the two have differentiated into independent species [14]. Chloroplast fragments indicate that *L. traubii* belongs to a clade closely related to populations in eastern China within the *L. aurea* complex. Therefore, *L. traubii* should be regarded as one of the varieties of *L. aurea*, and *L. aurea* is a typical aneuploid complex containing multiple varieties [24].

According to previous studies, research on *L. aurea* has mainly focused on morphology, cytology, physiology, pharmacology [24,25,26,27,28], with relatively little study on its distribution patterns and evolution. Under the context of climate change, studying the evolutionary process of the *L. aurea* complex across geological historical stages using species distribution models is critical for determining its mode of origin and population dispersal. In this study, we performed ecological niche modeling analyses: (1) to reconstruct the historical migration trends of the *L. aurea* complex since the Last Interglacial, (2) to identify the potential migration corridors supported by genetic evidence, and (3) to assess the divergent 103 responses of different cytotypes to future climate change. These findings will provide critical insights for the conservation of *Lycoris* germplasm in East Asia. Understanding these dynamics provides critical insights for the conservation and adaptive management of *L. aurea* under global climate change.

## 2. Results

### 2.1. Model Parameter Optimization and Simulation

After filtering, a total of 132 species occurrence points and 7 environmental variables (bio2, bio3, bio9, bio12, bio14, bio15, bio19) were retained (Supplementary Tables S1 and S2). These occurrence points were mainly concentrated in Yunnan, Guangxi, Guangdong, Guizhou, Sichuan, Chongqing, Hubei, Hunan, Taiwan of China and the Ryukyu Islands of Japan (Figure S1 in Supplementary Materials). Cross-validation results of the regularization multiplier (RM) and feature combination (FC) parameter combinations using the ENMeval package (version 2.0.4) showed that when delta.AICc was 0, the optimal combination was an RM of 1 and an FC of H (Figure S2 in Supplementary Materials).

**Figure 1:**
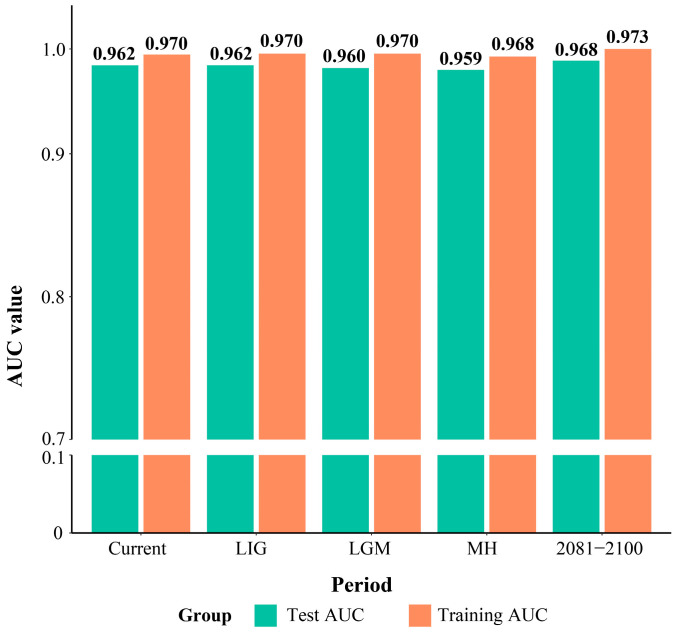
Training and test AUC values of model simulations across different periods.

Based on MaxEnt (version 3.4.4) simulations of the *L. aurea* complex across different periods, both the training AUC and test AUC values exceeded 0.9 after 10 repetitions (Figure 1, Supplementary Table S3). According to the AUC evaluation criteria, the model exhibited high predictive accuracy and good reliability for the *L. aurea* complex across different periods. Combined with field surveys and literature reports, the actual distribution range of *L. aurea* also showed a high degree of consistency with the highly and moderately suitable distribution areas in the current period. In the model training results, the 10th percentile threshold for the current period was 0.29. Referring to this threshold, 0.3 was set as the critical point distinguishing low suitability from moderately high suitability: highly suitable distribution areas were set to 0.5–1, moderately suitable distribution areas to 0.3–0.5, lowly suitable distribution areas to 0.1–0.3, and unsuitable distribution areas to 0–0.1.

**Figure 2:**
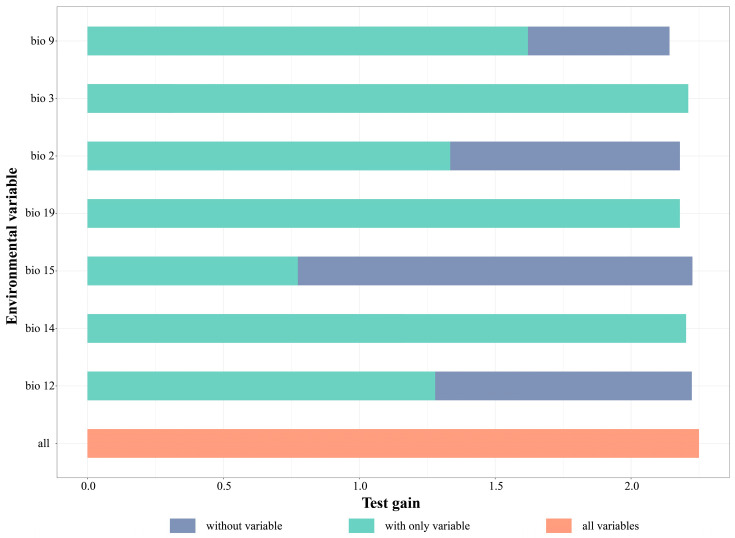
The Jackknife test result of current environmental factor for *Lycoris aurea* under three conditions: modeling without the variable, with only the variable, and with all variables.

The results of the jackknife analysis showed that the most influential factors on the potential distribution areas of *L. aurea* were primarily bio9 (mean temperature of the driest quarter), bio2 (mean diurnal range), bio12 (annual mean precipitation), and bio14 (precipitation of the driest month). Among these, bio12, bio14, and bio9 contributed the most and had the highest permutation importance, with a cumulative contribution of 86.1% and cumulative permutation importance of 72%. When using a single environmental variable for model prediction, the bio9 factor had the highest importance in test gains and normalized training gains, with the highest AUC value, making it the most influential environmental variable on the distribution of the *L. aurea* complex populations (Figure 2).

**Figure 3:**
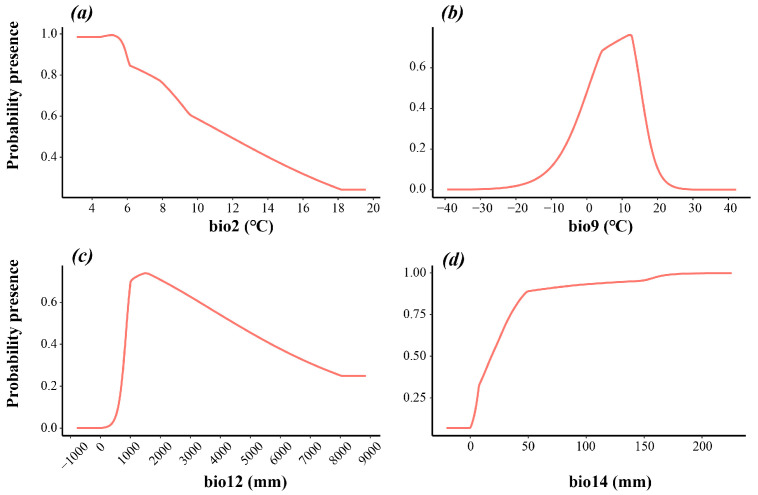
Suitable value ranges of the four main factors for *Lycoris aurea*. (**a**): bio2; (**b**): bio9; (**c**): bio12; (**d**): bio14.

Additionally, the MaxEnt model generated response curves for each factor, showing the change in the predicted probability of occurrence with variation in that climatic variable, while holding all other variables at their average sample values (Table 1). According to the response curves for climatic variables, *L. aurea* is suited to environments where the mean temperature of the driest quarter ranges from 0.5 to 15 °C, the mean diurnal range is 0 to 8.6 °C, annual mean precipitation is 900 to 4500 mm, and the precipitation of the driest month is not less than 20 mm (Figure 3).

### 2.2. Distribution Predictions of Different Periods

**Figure 4:**
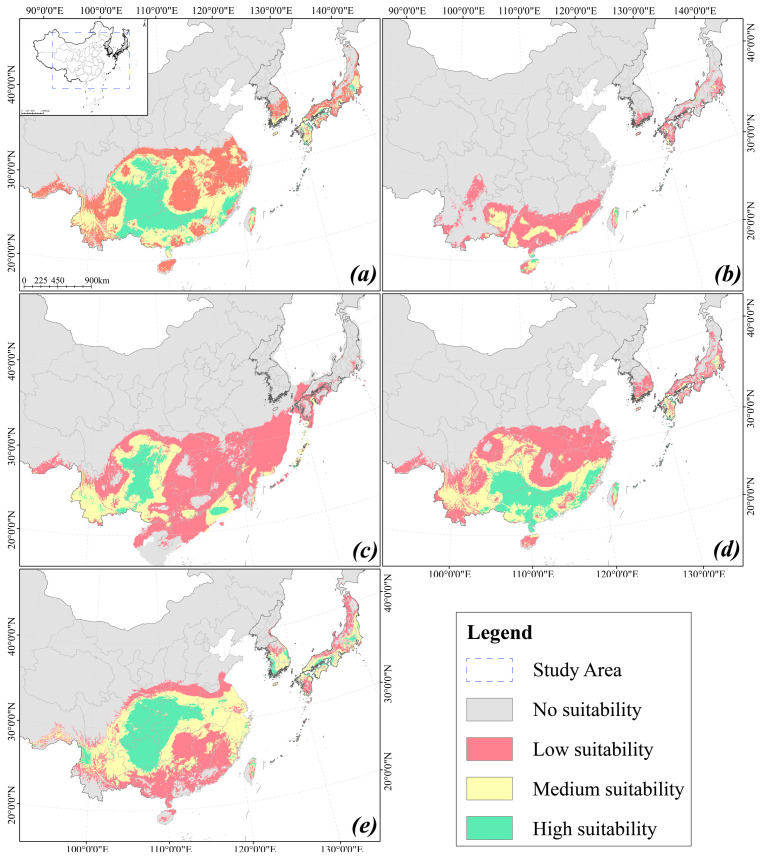
Potential distribution patterns of the *L. aurea* complex during different historical periods and in the future. Colors represent varying degrees of habitat suitability: Grey indicates Unsuitable (0–0.1), while Red is used for Low suitability (0.1–0.3), Yellow for Medium suitability (0.3–0.5), Green for High suitability (0.5–1.0). Overview of the study area in East Asia is displayed at the top left corner of panel (**a**); the dashed line box indicates the magnified region shown in panels (**a**–**e**). (**a**) Potential distribution for the Current period. (**b**) Potential distribution for the LIG (Last Interglacial) period. (**c**) Potential distribution for the LGM (Last Glacial Maximum) period. (**d**) Potential distribution for the MH (Mid-Holocene) period. (**e**) Projected potential distribution for the 2081–2100 period.

The MaxEnt model showed that in the current period, the suitable distribution area of high, medium and low levels of the *L. aurea* complex in East Asia accounts for 5.74%, 8.44%, and 10.12% (Table 2, Figure 4a). Medium to highly suitable distributions are primarily located in central, southern, southwestern, southeastern coastal China, northern Taiwan province, as well as Kyushu Island, the Seto Inland Sea coast, Kanto Plain, and Ryukyu Islands in Japan. Highly suitable areas include Sichuan, Chongqing, Hubei, Guizhou, Hunan, Yunnan, Guangxi, Guangdong, Fujian, Jiangxi, Taiwan, and some areas in Japan.

For different historical periods, the MaxEnt model simulation shows that during the interglacial period, the suitable distribution area of the *L. aurea* complex is 7.939 × 10^5^ km^2^, which is 68.1% less than the current period. It mainly concentrated in Guangdong, Guangxi, Hainan, Guizhou, Fujian, Yunnan, Sichuan, Taiwan, and the Ryukyu Islands (Figure 4b). During the LGM, the suitable habitats underwent a large-scale expansion. Populations in mainland China extended westward, while southeastern coastal populations migrated to the edge of the exposed continental shelf. In contrast, suitable habitats on islands such as Taiwan, Hainan, and the Ryukyu Islands contracted (Figure 4c). The total area of the suitable area had reached 2.6262 × 10^6^ km^2^. During the Mid-Holocene, the East China Sea land bridge submerged, reducing suitable distribution areas, but overall showing an expansion trend. The total area suitable for distribution is 2.3293 × 10^6^ km^2^, mainly distributed in Guizhou, Guangxi, Guangdong, Fujian, Taiwan, Yunnan, Chongqing, and southern regions of the Kyushu Island (Figure 4d).

Under continued global warming, the suitable habitats for *L. aurea* are projected to undergo a gradual northward shift and expansion during the period 2081–2100. Compared to the current period, the medium- and high-suitability habitats are projected to increase by 19.51% and 6.64%, respectively, resulting in a total area gain of 1.91 × 10^5^ km^2^. However, concurrent with this overall northward expansion, the northern and southern range margins in mainland China are also predicted to migrate northward. The southern margin is expected to retreat from South China to southern Guizhou Province, while the northern margin is projected to advance to the area south of the Yellow River. Furthermore, the suitable habitats in Taiwan Province will contract towards the northeast. This northward expansion of medium- and high-suitability habitats will be most pronounced in regions such as South Korea and Japan (Figure 4e).

### 2.3. Analysis of Centroid Shifts in Suitable Habitats and Migratory Paths

**Figure 5:**
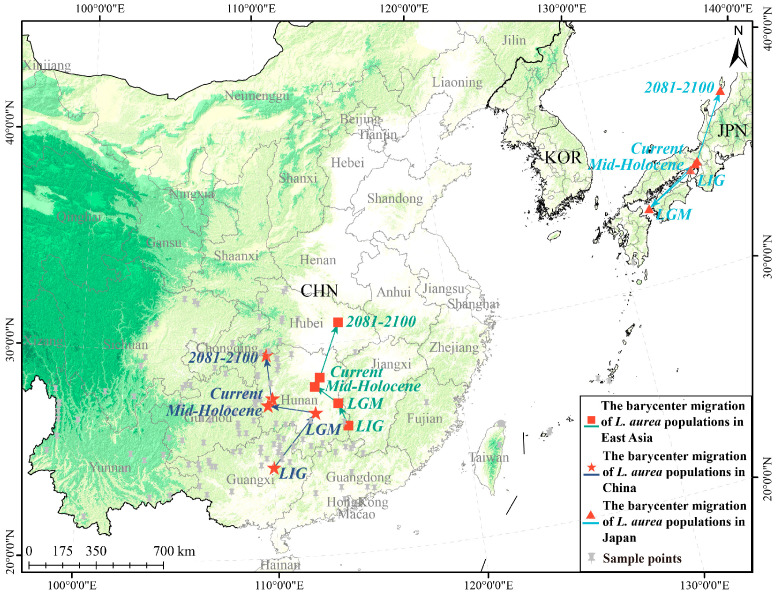
Shifts in the habitat suitability centroid for the *Lycoris aurea* complex across different time periods. Different shapes denote the centroids for specific geographic regions: squares represent the centroid for populations in Japan, pentagrams (stars) represent the centroid for populations in China, and triangles represent the overall centroid for the entire East Asia region. The lines connecting the centroids illustrate the direction of migration between periods.

Model projections indicate that the centroid of suitable habitats for the *L. aurea* complex in the Sino-Japanese region has been gradually shifting northward since the LIG. In China, the centroid migrated from Guangxi to Hunan and is projected to move further northwestward in the future. In Japan, it shifted from the northern shore of the Seto Inland Sea to southern Kyushu, with a potential future migration towards Hokkaido (Figure 5).

**Figure 6:**
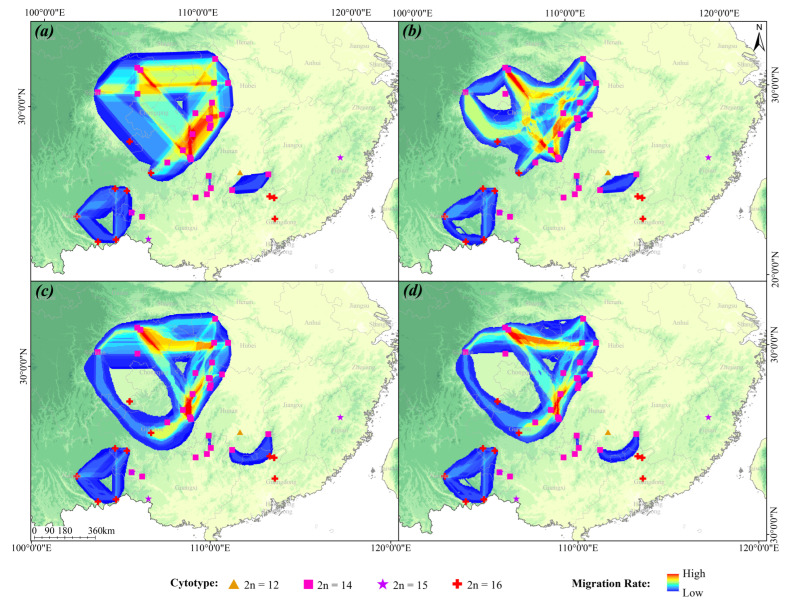
Migration pathways of the *Lycoris aurea* complex in different historical periods. (**a**) Migration pathways during the LIG period. (**b**) Migration pathways during the LGM period. (**c**) Migration pathways during the MH period. (**d**) Migration pathways during the Current period. The color gradient indicates migration probability, increasing from blue (low probability) to red (high probability). Different shapes denote specific cytotypes: triangles represent Cytotype I (2n = 12), while squares for Cytotype II (2n = 14), stars for Cytotype III (2n = 15), and cruciate spots for Cytotype IV (2n = 16).

Migration path analysis revealed multiple migration corridors for the *L. aurea* complex since the Last Interglacial (LIG), primarily concentrated in: (1) Central and Southwest China (including Sichuan, Chongqing, Guizhou, Hubei, and Hunan); (2) the mountainous regions of eastern Yunnan; and (3) the Nanling Mountains region at the junction of Hunan, Guangxi, and Guangdong (Figure 6).

Among these, the Central and Southwest China corridor exhibited the highest migratory connectivity, predominantly involving populations of cytotype II (2n = 14) and cytotype IV (2n = 16). The other two migration paths were dominated by intra-cytotype migration; the Yunnan Plateau corridor was characterized by a lower migration rate, while the migration paths in the Nanling Mountains region were comparatively short.

### 2.4. Potential Distribution Modeling and Divergence Analysis for the Two Major Cytotypes

**Figure 7:**
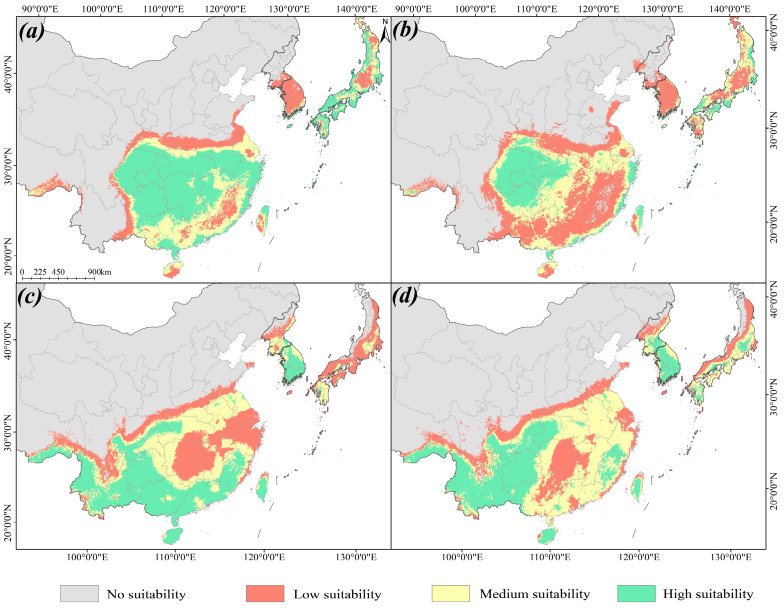
Potential distributions of the two major cytotypes (*Lycoris aurea*; 2n = 14 and 2n = 16) for the current and future (2081–2100) periods. (**a**,**b**) Potential distribution of Cytotype II (2n = 14) for the current (**a**) and future (**b**) periods. (**c**,**d**) Potential distribution of Cytotype IV (2n = 16) for the current (**c**) and future (**d**) periods. Different colors indicate levels of habitat suitability: Grey indicates Unsuitable (0–0.1), while Red for Low suitability (0.1–0.3), Yellow for Medium suitability (0.3–0.5), Green for High suitability (0.5–1.0).

The simulation results indicate that under the current period, the medium-to-high suitability habitats for Cytotype II are primarily located in large parts of China south of the Qinling-Huaihe Line and east of the Hengduan Mountains. Northern Taiwan province and the Ryukyu Islands also exhibit high suitability. Additionally, highly suitable areas are found in the coastal regions of Japan south of Hokkaido, including the islands of Kyushu, Shikoku, and Honshu (Figure 7a). In contrast, the medium-to-high suitability habitats for Cytotype IV are mainly distributed across China’s Yunnan-Guizhou Plateau, Guangdong, Guangxi, Fujian, Hainan, and Taiwan, as well as the Ryukyu Islands and most parts of South Korea (Figure 7c).

Under future climate warming scenarios, the suitable habitats for Cytotype II are projected to undergo a large-scale contraction. The medium-to-high suitability habitats are projected to retreat to regions including Chongqing, eastern Sichuan, northern Guizhou, and coastal areas south of the Yangtze River Delta in China. Suitable habitats in Taiwan and the Ryukyu Islands are expected to remain relatively stable. In Japan, these habitats are projected to contract towards southern and eastern Honshu and Shikoku islands (Figure 7b). For Cytotype IV, medium-to-high suitability habitats are projected to contract in areas such as Fujian, Guangdong, and Guangxi in China. However, they are predicted to show an expansion trend in eastern Japan, south of Fukushima, with the overall range of medium-to-high suitability habitats in Japan expanding considerably (Figure 7d).

**Figure 8:**
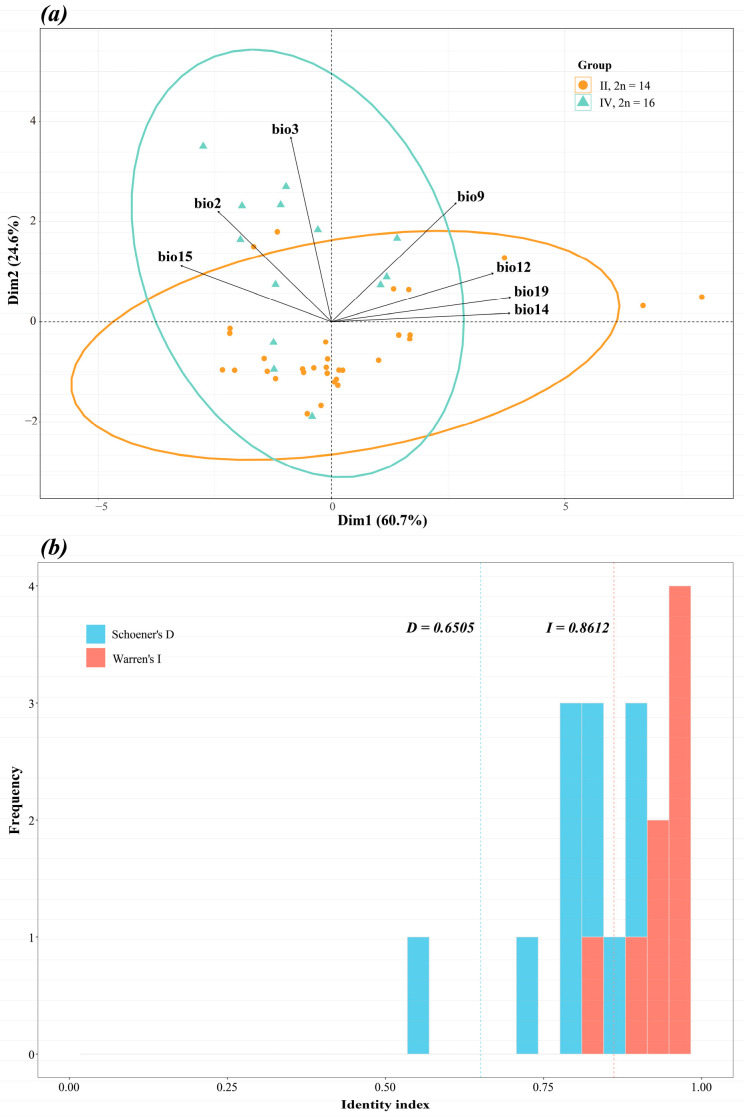
Principal Component Analysis (PCA) and identity test for the two major cytotypes (*Lycoris aurea*; 2n = 14 and 2n = 16). Different colors represent the two cytotypes. (**a**) Results of the PCA. (**b**) Results of the identity test.

Principal Component Analysis (PCA) results revealed a degree of separation between the cytotype II and IV populations (Figure 8a). The first two principal components (PCs) explained 60.7% and 24.6% of the total variance, respectively. This separation was primarily driven by bio3, bio12, bio14, and bio19. Three precipitation-related environmental variables (bio12, bio14, and bio19) exerted the strongest influence on the Cytotype II (2n = 14) populations, showing a significant positive correlation. In contrast, the temperature-related variable bio3 had the strongest influence on the Cytotype IV (2n = 16) populations, also exhibiting a significant positive correlation.

Results from the niche equivalency test showed that for both Schoener’s D and Warren’s I, the observed niche overlap values fell within the 95% confidence interval of the null distribution (Figure 8b). This indicates that the null hypothesis of niche equivalency could not be rejected, suggesting that the niches of the two cytotypes have not significantly diverged and are not statistically different.

## 3. Discussion

### 3.1. Spatio-Temporal Dynamics and Phylogeographic History of the Lycoris aurea Complex

The predictive performance of our model for the current period was corroborated by our multi-year field survey data, which demonstrates a high degree of accuracy. Leveraging this validated model, we projected the distribution of the *L. aurea* complex onto past (LIG, LGM, and Mid-Holocene) and future climatic scenarios (Figure 4). Our paleodistribution modeling suggests that the complex underwent a significant range expansion from the LIG to the LGM. This “glacial expansion” pattern, while contrary to the typical “glacial contraction” hypothesis seen in many European and North American taxa, aligns with findings for other East Asian plants [11,29]. Subsequently, during the Mid-Holocene climatic optimum, warmer and moister conditions likely facilitated further expansion, with suitable habitats gradually shifting to form the distribution observed today. In the future, concurrent with projected global warming trends, the suitable habitats for the complex are predicted to undergo a pronounced northward shift—a common pattern anticipated for many plant species [30,31]. However, our results reveal a critical divergence in regional responses: populations in mainland China are projected to further expand their range, whereas those on Taiwan Island and the Ryukyu Islands are predicted to experience significant range contraction. This projected contraction is a direct consequence of island biogeographic constraints, which inherently limit species’ ranges. This phenomenon is analogous to the predicted trends for narrow-range specialist species in northern China, which are similarly identified as being highly vulnerable to climatic shifts [31].

Islands typically act as significant biogeographic barriers, impeding gene flow and dispersal [32,33]. Conversely, sea-level regressions during glacial periods expose continental shelves, creating transient “land bridges” that can transform these barriers into dispersal corridors. The Bering Land Bridge (connecting Asia and North America) and the East China Sea (ECS) Land Bridge (connecting China, Japan, and Korea) are classic examples. During glacial maxima, these exposed corridors are known to have facilitated the migration of numerous plant taxa, such as *Podophyllum*, *Diphylleia*, and *Machilus* [5,8]. However, our findings for *Lycoris aurea* present a notable exception to this paradigm. Our SDM projections indicate that the exposed ECS Land Bridge offered low habitat suitability for the complex during the LGM, suggesting it did not function as an effective corridor (Figure 4d). Concurrently, our independent modeling for Cytotype II showed that its populations on Taiwan Island and the Ryukyu Islands maintained consistently high habitat suitability across all historical periods. This suggests these islands did not merely act as barriers, but rather as persistent “insular refugia”, characterized by strong independence and long-term population stability, even when a “bridge” was theoretically present. This could be due to the environmental heterogeneity of the ECS land bridge, or it might be that the divergence predated the emergence of the ECS land bridge [34,35].

Previous genetic studies also demonstrated that the *L. aurea* complex is polyphyletic, comprising multiple independent evolutionary lineages [24]. Building on these established findings, we hypothesize that these insular populations (Taiwan and Ryukyu) constitute one such distinct lineage. The post-glacial sea-level rise in the ECS, which submerged the shelf, acted as a decisive vicariant event that enforced this isolation and catalyzed their independent evolutionary trajectories. This pattern of island-driven vicariance is not unique; the divergence time between *Cardiocrinum cathayanum* (China) and *C. cordatum* (Japan) coincides with the initial fragmentation of the Japanese archipelago from the Eurasian continent [11]. This strongly suggests that the formation of these islands (Taiwan, Ryukyu, and Japan) acted as a powerful barrier to gene flow, driving the formation of distinct lineages. Otherwise, our observation that the *L. aurea* complex primarily dispersed along least-cost paths—tracking river systems and mountain ranges—indirectly reinforces the critical role of topographical, hydrological, and oceanic barriers in shaping the complex’s phylogeographic structure [36,37,38].

### 3.2. Differential Impacts of Climatic Factors on Lycoris aurea Cytotypes

For the *L. aurea* complex as a whole, the primary limiting environmental factors identified by the model were bio9, bio2, bio12, and bio14, with bio9 and bio12 exerting the strongest influence. This modeled macro-distribution aligns closely with our field observations, which confirm the main distribution is south of the Qinling-Huaihe Line, with high concentrations in the mountainous regions of Southwest China and the Lingnan hills, particularly in Guizhou, Guangxi, and Yunnan provinces. Furthermore, the divergence of functional traits observed across different geographic distributions underscores that climate-dominated environmental factors are key selection pressures imposed on plants. This well-established evolutionary driver has been confirmed in numerous species to drive adaptive differentiation in the morphology of organs such as roots, stems, leaves, and flowers [39,40]. As demonstrated in the previous morphological analysis [24], the *L. aurea* complex is a clear exemplar of this phenomenon; its Cytotype II and Cytotype IV populations exhibit significant morphological differences in key leaf functional traits, such as leaf thickness, stomatal density, stomatal index, and spongy tissue thickness. As the primary organs for photosynthesis and transpiration, leaves are highly responsive to environmental change, and numerous studies have confirmed the strong correlation between leaf functional traits and environmental factors [39,41,42,43].

Crucially, we detected significant climatic differences among the cytotypes. bio9, in particular, was a key differentiating factor between Cytotype II vs. IV. This ecological distinction is reflected in their geography: Cytotype II is a widespread generalist across East Asia, whereas Cytotype IV is a regional specialist restricted to southern and southwestern China. Although their ranges broadly overlap, they exhibit clear parapatric patterns in certain regions. The Yunnan-Guizhou Plateau, for example, is dominated by Cytotypes III and IV, while Cytotype II is rare (Figure S1). We hypothesize this segregation is linked to the plateau’s unique climate, characterized by high humidity (high bio12/14) and a low mean diurnal range (low bio2). Our PCA results substantiate this hypothesis. Cytotype II was most strongly influenced by precipitation variables (bio12, bio14, bio19), whereas Cytotype IV was most strongly influenced by a temperature variable (bio3). This aligns with extensive research identifying temperature and precipitation as the key elements governing plant niche differentiation and distribution, particularly in the context of cytotype evolution [44,45].

### 3.3. The “Filter Corridor” Hypothesis for the LGM East China Sea Land Bridge and Genetic Connectivity

During the Quaternary glacial cycles, sea-level fluctuations led to the recurrent formation and disappearance of the ECS land bridge, which had a crucial impact on the recent lineage divergence and population dynamics of the East Asian flora [35,46,47]. Genetic studies indicate a close genetic affinity between *L. aurea* populations in Japan and those in eastern coastal China, suggesting a historical corridor for gene flow must have existed [24]. However, our LGM simulation results provide a key, but seemingly contradictory, mechanistic explanation for this genetic pattern. Our model shows that while the exposed ECS land bridge did connect mainland China, Taiwan, and the southern Japanese islands during the LGM, its overall habitat suitability was very low.

This finding is highly consistent with the paleoenvironmental heterogeneity proposed by Qiu et al. (2011), who suggested the LGM land bridge contained arid, non-forest patches that impeded the dispersal of forest-adapted species [35]. If *L. aurea* was tolerant of such open environments, it is plausible that it crossed this bridge via “stepping-stone” dispersal or in a low-density pattern. This model of a “filter corridor,” rather than a broadly suitable “superhighway,” reconciles both observations: it explains the genetic connectivity and simultaneously, explains why our model shows widespread low suitability.

### 3.4. Limitations and Future Perspectives

Currently, there is no consensus on the precise origin of the *Lycoris aurea* complex. Existing studies have been largely limited to using chloroplast genome (cpDNA) fragments or karyotype analysis to infer its origin patterns and ancestral populations. Although whole-genome sequencing and chromosome-level assembly technologies have demonstrated immense potential in reconstructing complex evolutionary histories [11,48,49], accurately deciphering the reticulate evolutionary history of the *L. aurea* complex remains challenging due to its exceptionally large genome size and complex karyotype evolution [50]. Furthermore, the extreme scarcity of fossil records for Lycoris and its closely related species limits our ability to accurately calibrate historical divergence times.

Despite these limitations, our SDM results provide critical clues regarding its origin. We identified multiple independent glacial refugia for *L. aurea* during LGM, such as the mountainous regions of Southwest China, Taiwan Island, and the Ryukyu Islands. This finding corroborates the genetic evidence from Wang et al. (2022), collectively supporting the hypothesis that the *L. aurea* complex may have undergone a “polytopic origin,” with China being the most likely primary center of origin [24]. In the future, precisely resolving its exact mode of origin and complex evolutionary trajectory urgently requires the construction of a high-quality, chromosome-level reference genome, combined with in-depth analyses using large-scale population whole-genome resequencing data.

## 4. Materials and Methods

### 4.1. Datasets

#### 4.1.1. *Lycoris aurea* Complex Distribution Dataset

The distribution data of *L. aurea* were primarily sourced from prior field surveys and digital specimen searches on the Web, yielding a comprehensive collection of population distribution points. Field sampling contributed 46 *L. aurea* population locations; additional records (1591 entries) were gathered from literature reviews and searches on digital platforms such as the Chinese Virtual Herbarium (CVH, https://www.cvh.ac.cn/), the Global Biodiversity Information Facility (GBIF, https://www.gbif.org/, https://doi.org/10.15468/39omei accessed on 10 April 2025), iNaturalist (https://www.inaturalist.org/), and the Japanese Natural History Museum specimen database (https://science-net.kahaku.go.jp/).

To ensure model accuracy, the collected *L. aurea* complex population distribution points were manually screened. The duplicate and undefined (without definite coordinates) records were removed from the data set at first. Based on the information gathered through prior field investigations on the genus *Lycoris*, we have determined that certain *L. aurea* specimen records catalogued of the CVH from the provinces of Jiangsu, Shanghai, Zhejiang, and Anhui in fact only document the distribution of *L. chinensis*, with no evidence of extant wild populations of *L. aurea*, these potentially misidentified and uncertain points are also removed. Furthermore, some points where specimen descriptive information does not match the characteristics of *L. aurea.* The remaining points were imported into ArcGIS v10.8 to detect geographic distance between points. Considering the climate data accuracy, only one distribution point per grid cell was retained to prevent model overfitting, discarding closely located adjacent data. A final dataset of occurrence records have added in Supplementary Table S1.

#### 4.1.2. Environmental Dataset

The climate data used in this study were sourced from the WorldClim database (https://www.worldclim.org/) [51], encompassing five time periods: current climate (1970–2000), Last Interglacial (LIG, ~12–14 ka B.P.), Last Glacial Maximum (LGM, ~2.2 ka B.P.), Mid-Holocene (MH, approximately 6000 years ago), and future (2081–2100). The resolution was 2.5 min. LIG and LGM climate data were derived from CCSM4 model simulations, while future climate projections used the BCC-CSM2-MR model from CMIP6, under the SSP370 shared socioeconomic pathway. Elevation data were obtained from SRTM (https://srtm.csi.cgiar.org) with a 30 m resolution.

The regional maps of China for the model were sourced from the 1:1 million scale public edition basic geographic information data published by the National Geographic Information Resources Catalogue Service System (https://www.webmap.cn/). Maps of Japan, Korea, and North Korea were derived from the Global Administrative Areas database (GADM, https://gadm.org/).

To avoid model overfitting due to the 19 climate variables’ primary relation to temperature and precipitation and their correlation with elevation, Pearson correlation analysis was conducted using R packages ‘ENMTools’ (version 1.1.7) and ‘usdm’ (version 2.1.7) [52]. Variables with a correlation coefficient greater than 0.7 and a variance inflation factor (VIF) higher than 10 were excluded, and the remaining variables were used for model simulation.

### 4.2. Statistical Analysis

#### 4.2.1. MaxEnt Model Parameter Optimization

Based on the filtered *L. aurea* complex distribution data and climate variables, the ENMeval R package (version 2.0.4) was used to optimize the regularization multiplier (RM) and feature combination (FC) parameters of the MaxEnt model, aiming to identify the best parameter combination for simulation [53]. The FC parameter was configured based on the combination of five fundamental feature categories: linear (L), quadratic (Q), hinge (H), product (P), and threshold (T). In this study, RM values ranged from 0.5 to 5, increasing by 0.5 each time, and eight feature combinations were tested: L, LQ, LQH, LQHP, LQHPT, H, HPT, QPT. ENMeval performed parameter combination tests, assessing model complexity under different parameters using the AIC criterion. The lower the delta.AICc value, the better the model fit, with delta.AICc of 0 indicating the optimal parameter combination.

#### 4.2.2. Model Construction and Accuracy Assessment

The filtered distribution data and climate variables were imported into MaxEnt version 3.4.4 software [54]. Seventy-five percent of the distribution points were randomly selected for training the model, while 25% were used for testing. The maximum iteration was set to 1000 with 10 repetitions. The model FC and RM were set according to the results of ENMeval, with other values at default. The current distribution of *L. aurea* was modeled, followed by projecting this model onto past and future climate layers to reconstruct potential suitable distribution areas during the LGM, LIG, Mid-Holocene, and the 2081–2100 period under the SSP370 scenario.

Model accuracy and reliability were evaluated based on the area under the Receiver Operating Characteristic (ROC) curve (AUC). AUC values range from 0 to 1, with higher values indicating better model fit and credibility. AUC values of 0.9 to 1.0 indicate excellent fit, 0.8 to 0.9 good fit, 0.7 to 0.8 moderate fit, and below 0.7 poor fit. Finally, the results with good AUC fitting were imported into ArcGIS v10.8 (ESRI, https://www.esri.com/) for reclassification of habitat suitability. Based on the 10th percentile training presence threshold, the simulated results were divided into highly suitable, moderately suitable, lowly suitable, and unsuitable distribution areas.

#### 4.2.3. Migration and Dispersal Pathway Analysis

The area and centroid changes in potential suitable distribution areas across different periods were calculated using SDMToolbox (http://www.sdmtoolbox.org) [55]. Considering oceanic barriers and land bridge connectivity during glaciations, discussions on suitable distribution area centroid shifts in China and Japan were focused primarily on mainland China, Hainan Island, Taiwan, and the four main Japanese islands.

Based on the chloroplast DNA haplotype information of 42 *L. aurea* complex populations analyzed by Wang (2022) [24], combined with the model-predicted potential distribution results, the SDMToolbox’s least-cost path analysis tool was used to calculate and analyze migration and dispersal pathways of *L. aurea* since the Last Interglacial. It was assumed that higher suitability areas incur lower species migration and dispersal costs. The potential distribution models from the Last Interglacial to present were converted into habitat resistance layers (1-SDM), overlaid with species haplotype information, to calculate the least-cost migration paths between populations sharing haplotypes across different historical periods.

#### 4.2.4. Differential Analysis of the Two Dominant Cytotype Populations

Cytological studies on *L. aurea* have shown that cytotype II (2n = 14) and cytotype IV (2n = 16) are the dominant cytotypes within the complex, with relatively few reports on populations of other cytotypes. Therefore, for these two dominant cytotype populations, we separately simulated their current and future potential suitable distribution areas, with model construction parameters and climatic variables referring to the optimized results mentioned earlier. Meanwhile, variables used for simulation in the distribution regions of cytotype II and cytotype IV populations were extracted using the R package ‘raster’ for PCA, to compare the correlation between climatic differences and their distribution impacts. Additionally, we tested the niche consistency between the two cytotypes using the ‘ENMtools’ package to determine whether niche differentiation has occurred [52].

All the aforementioned analytical operations using R packages were performed in R (version 4.5.1, https://www.r-project.org/).

## 5. Conclusions

Based on the MaxEnt model, this study reconstructed the potential suitable habitats and spatiotemporal migration patterns of the *L. aurea* complex across different historical periods. Since its origin, the complex has primarily flourished in regions south of the Yangtze River in China, as well as in Kagoshima Prefecture and the Ryukyu Islands in Japan. During the LGM, multiple glacial refugia likely existed, notably in Southwest China, coastal Guangdong, Taiwan Island, the Ryukyu Islands, and Kagoshima. Following the post-glacial period, the complex expanded from these refugia to establish its current distribution pattern. Temperature and precipitation are the dominant environmental factors shaping the distribution of the *L. aurea* complex. Specifically, divergence between dry and wet climates have driven the differentiation of cytotypes and the adaptive evolution of morphological structures. However, due to the limited availability of extensive population sampling and comprehensive chromosome-level genomic evidence, accurately estimating the divergence times of different cytotypes remains challenging. Consequently, our inferences regarding specific glacial refugia and historical migration trends still hold certain limitations. Therefore, to gain deeper insights into the origin and evolutionary history of the *L. aurea* complex, future studies should prioritize broader population sampling—particularly from insular regions like Taiwan province and the Ryukyu Islands—and employ advanced multi-omics approaches, including genomics, transcriptomics, and cytomics.

## Figures and Tables

**Table 1:** Percent Contribution, Permutation Importance, and Suitable Range of seven Environmental Variables for *Lycoris aurea*.

Environment Variables	Percent Contribution	Permutation Importance	Suitable Range
bio2	4.4	4.9	31.3~118.43 °C
bio3	4.8	7.3	17.81~87.4%
bio9	20.4	22.6	5.0~152 °C
bio12	35.9	35.5	879.9~4464.1 mm
bio14	29.8	13.9	18.36~225.5 mm
bio15	1.3	4.3	0.59~21.3%
bio19	3.4	11.6	−297.6~177.37 mm

**Table 2:** Suitable distribution area of Lycoris aurea for different periods.

Period	Suitable Distribution Area (×10^4^ km^2^)			
	Low Suitable	Medium Suitable	High Suitable	Total
Current period	103.60	86.30	58.71	248.61
LIG	62.46	15.65	1.28	79.39
LGM	175.23	62.42	24.97	262.62
MH	119.89	72.18	40.87	232.93
2081–2100	101.96	103.13	62.61	267.69

## Data Availability

Data are contained within the article and Supplementary Materials. The distribution data of sampling sites used in this study are all derived from public datasets and literature reports, and the data sources have been listed in the text.

## Supplementary Materials

The following supporting information can be downloaded at https://www.mdpi.com/article/10.3390/plants15020272/s1, Figure S1. Map of the distribution of 132 filtered populations of the *Lycoris aurea* complex; Figure S2. AICc (left panel) and delta.AICc (right panel) of MaxEnt models (optimized via the ENMeval package) across varying regularization multipliers (RM); Table S1. Number of occurrence points of the *Lycoris aurea* complex collected from different databases and details of the filtering process; Table S2. Environment variables for MaxEnt modeling; Table S3. Area under the receiver operating characteristic curves (AUC, mean ± SD) of Maxent models in predicting the distribution of *Lycoris aurea* under different periods.

## Abbreviations

The following abbreviations are used in this manuscript:

SDM	Species distribution model
LIG	Last Interglacial (LIG, ~12–14 ka B.P.), Last Glacial Maximum
LGM	Three letter acronym
MH	Mid-Holocene
RM	Regularization multiplier, L (liner), Q (quadratic), H (hinge), P (product), T (threshold)
FC	Feature combination
ROC	Receiver Operating Characteristic
AUC	Area Under the ROC Curve
PCA	Principal Component Analysis
ECS	East China Sea
