# Supplementary material for: Evolutionary Patterns Under Climatic Influences on the Distribution of the Lycoris aurea Complex in East Asia: Historical Dynamics and Future Projections
Source: Plants (Basel). 2026 Jan 16;15(2):272. doi: 10.3390/plants15020272 (PMC12845144; doi:10.3390/plants15020272)
Supplement: Supplementary file 1 [file plants-15-00272-s001.zip › plants-4006333-supplementary.pdf]

Supplementary Files

Figure S1. Map of the distribution of 132 filtered populations of the *Lycoris aurea* complex. Different colors and shapes correspond to specific chromosome numbers reported in the literature or identified in our prior research. **Legend:** Green triangle: 2n = 12; Orange square: 2n = 14; Purple cross: 2n = 15; Red star: 2n = 16; Blue circle : Populations with unreported cytotypes.

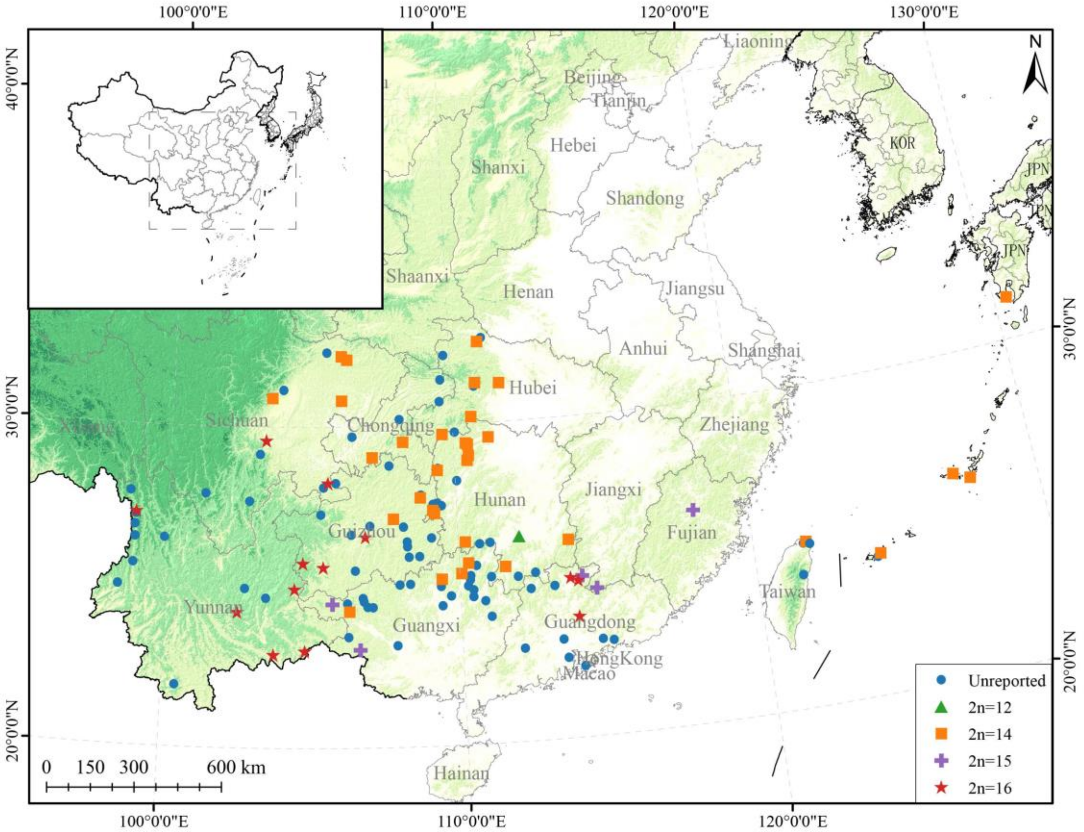

Figure S2. AICc (left panel) and delta.AICc (right panel) of MaxEnt models (optimized via the ENMeval package) across varying regularization multipliers (RM). Abbreviations for MaxEnt feature types: L = Linear, Q = Quadratic, H = Hinge, P = Product, T = Threshold. Colored lines correspond to distinct feature combinations: Orange (FC) = [add specific composition, e.g., predefined base feature combination]; Red (H) = hinge-only feature; Yellow (L) = linear-only feature; Green (LQ) = linear + quadratic features; Teal (LQH) = linear + quadratic + hinge features; Blue (LQHP) = linear + quadratic + hinge + product features; Pink (LQHPT) = full feature set (linear + quadratic + hinge + product + threshold).

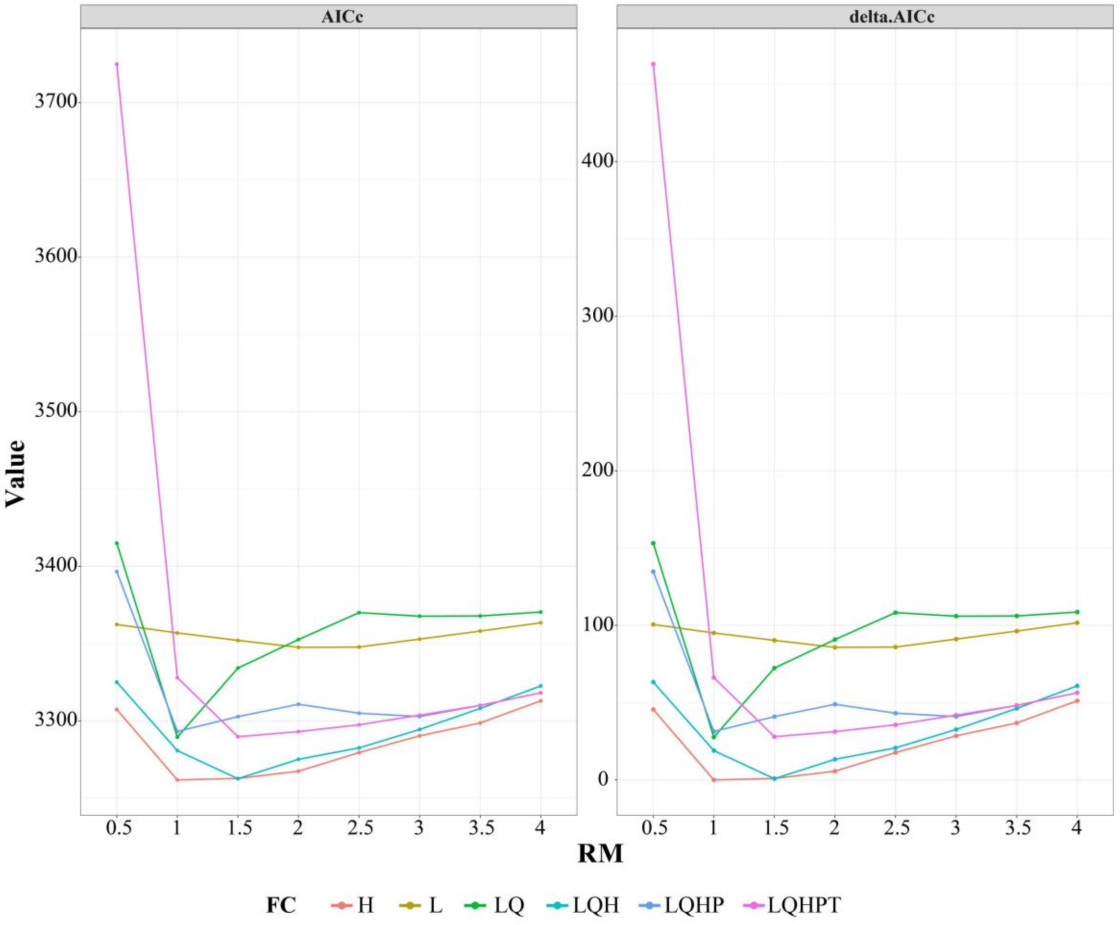

Table S1. Number of occurrence points of the *Lycoris aurea* complex collected from different databases and details of the filtering process.

<table>
<tr><th>Source（Total number of search results）</th><th>Number of remained points</th><th>Screening details</th></tr>
<tr><td>Field surveys（46）</td><td>45</td><td>Exclusion of similarly spaced points within the same raster image for climate variables（2.5min）</td></tr>
<tr><td>Chinese Virtual Herbarium（598）</td><td>43</td><td rowspan="4">First, eliminate points with duplicate records, no latitude and longitude, and latitude and longitude that do not match the information of the specimen collection site. Second, according to the information of specimen collection during the flowering and fruiting period, such as collection time and collection location, eliminate the doubtful points that may be identified as L. chinensis (L. chinensis, flowering in July-August, fruiting in September; L. aurea, flowering in August-September, fruiting in October). Than, Points of artificial introductions to cultivation. Finally, excluding the points with similar distance within the same raster image of 2.5m precision climate variables.</td></tr>
<tr><td>Global Biodiversity Information Facility（673）</td><td>31</td></tr>
<tr><td>Japanese Natural History Museum specimen database（41）</td><td>5</td></tr>
<tr><td>iNaturalist（233）</td><td>8</td></tr>
</table>

Table S2. Environment variables for MaxEnt modeling.

Environment Variable	Description	Unit
bio1	Annual Mean Temperature	℃
bio2*	Mean Diurnal Range (Mean of the monthly(max temp-min temp))	℃
bio3*	Isothermality (bio2/bio7) (× 100)	-
bio4	Temperature Seasonality (Standard deviation × 100)	-
bio5	Max Temperature of Warmest Month	℃
bio6	Min Temperature of Coldest Month	℃
bio7	Temperature Annual Range (bio5-bio6)	℃
bio8	Mean Temperature of Wettest Quarter	℃
bio9*	Mean Temperature of Driest Quarter	℃
bio10	Mean Temperature of Warmest Quarter	℃
bio11	Mean Temperature of Coldest Quarter	℃
bio12*	Annual Precipitation	mm
bio13	Precipitation of Wettest Month	mm
bio14*	Precipitation of Driest Month	mm
bio15*	Precipitation Seasonality (Coefficient of Variation)	-
bio16	Precipitation of Wettest Quarter	mm
bio17	Precipitation of Driest Quarter	mm
bio18	Precipitation of Warmest Quarter	mm
bio19*	Precipitation of Coldest Quarter	mm
alt	altitude	m

*: the factors retained for MaxEnt model simulation analysis after Person correlation detection.

Table S3. Area under the receiver operating characteristic curves (AUC, mean ± SD) of Maxent models in predicting the distribution of *Lycoris aurea* under different periods.

Period	AUC value	Standard Deviation
Current period	0.962	0.009
LIG	0.962	0.01
LGM	0.960	0.008
MH	0.959	0.005
2081-2100	0.965	0.018
